# Longitudinal Expression of Testicular *TAS1R3* from Prepuberty to Sexual Maturity in Congjiang Xiang Pigs

**DOI:** 10.3390/ani11020437

**Published:** 2021-02-08

**Authors:** Ting Gong, Weiyong Wang, Houqiang Xu, Yi Yang, Xiang Chen, Lijie Meng, Yongjian Xu, Ziqing Li, Sufang Wan, Qi Mu

**Affiliations:** 1 Key Laboratory of Animal Genetics, Breeding and Reproduction in The Plateau Mountainous Region, Ministry of Education, Guizhou University, Guiyang 550025, China; tgong@gzu.edu.cn (T.G.); wangxiaochui369@163.com (W.W.); yiyangnutrition@foxmail.com (Y.Y.); as.xchen2@gzu.edu.cn (X.C.); mlj18208544454@163.com (L.M.); xuyongjian0503@163.com (Y.X.); 2 Key Laboratory of Animal Genetics, Breeding and Reproduction, Guiyang 550025, China; 3 College of Animal Science, Guizhou University, Guiyang 550025, China; ZQli98@163.com (Z.L.); w18385462413@163.com (S.W.); Muqi0524@163.com (Q.M.)

**Keywords:** T1R3, testis, Congjiang Xiang pig, developmental biology

## Abstract

**Simple Summary:**

Taste receptor type 1 subunit 3 (T1R3), a sweet/umami taste receptor, is widely expressed from the tongue to the testis, and testis expression is associated with male sterility. In Congjiang Xiang pigs, T1R3 is expressed in elongating/elongated spermatids and Leydig cells in a stage-dependent manner during postnatal development and the spermatogenic cycle. T1R3 may contribute to regulation of spermatid differentiation and Leydig cell function, and may therefore help limit the incidence of various male reproductive pathologies.

**Abstract:**

Testicular expression of taste receptor type 1 subunit 3 (T1R3), a sweet/umami taste receptor, has been implicated in spermatogenesis and steroidogenesis in mice. We explored the role of testicular T1R3 in porcine postnatal development using the Congjiang Xiang pig, a rare Chinese miniature pig breed. Based on testicular weights, morphology, and testosterone levels, four key developmental stages were identified in the pig at postnatal days 15–180 (prepuberty: 30 day; early puberty: 60 day; late puberty: 90 day; sexual maturity: 120 day). During development, testicular T1R3 exhibited stage-dependent and cell-specific expression patterns. In particular, T1R3 levels increased significantly from prepuberty to puberty (*p* < 0.05), and expression remained high until sexual maturity (*p* < 0.05), similar to results for phospholipase Cβ2 (PLCβ2). The strong expressions of T1R3/PLCβ2 were observed at the cytoplasm of elongating/elongated spermatids and Leydig cells. In the eight-stage cycle of the seminiferous epithelium in pigs, T1R3/PLCβ2 levels were higher in the spermatogenic epithelium at stages II–VI than at the other stages, and the strong expressions were detected in elongating/elongated spermatids and residual bodies. The message RNA (mRNA) levels of taste receptor type 1 subunit 1 (T1R1) in the testis showed a similar trend to levels of T1R3. These data indicate a possible role of T1R3 in the regulation of spermatid differentiation and Leydig cell function.

## 1. Introduction

Taste sensations enable animals to evaluate which prospective foods are nutritious or toxic. Members of the taste receptor family 1 (T1Rs), encoded by *TAS1R* genes, T1Rs are largely responsible for sensing sweet and umami tastes in the taste buds. Taste receptor family 1 subunit 1 (T1R1)/taste receptor family 1 subunit 3 (T1R3) heterodimers form an umami taste receptor, and the taste receptor family 1 subunit 2 (T1R2)/T1R3 heterodimers recognize sweet tastes [1]. In taste cells, a heterotrimeric GTP-binding protein (gustducin)-mediated signaling cascade can be activated when T1Rs sense umami or sweet stimuli. A GTP-protein consisting of 2 subunits, α-gustducin (Gαgust) and βγ-gustducin (Gβγgust), activates phospholipase Cβ2 (PLCβ2) to increase intracellular Ca^2+^ levels. Gαgust is expected to activate phosphodiesterase (PDE), resulting in decreased intracellular cyclic adenosine monophosphate (cAMP) levels after ligand binding [1].

Although T1R3 was first detected in the tongue and contributes to the perception of acceptable foods, an increasing number of studies have reported the expression of T1R3 and its related signaling transduction cascade in non-taste tissues, including the digestive system, respiratory system, urinary bladder, pancreas, liver, brain, and testis [2].

There is evidence that testicular T1R3 may function in male sterility. For example, T1R3 is highly expressed in swine testicular tissues, especially in spermatogenic cells [3]. In mice, T1R3 and its related molecules, T1R1, G_αgust_, and taste receptor family 2 subunit 5 (T2R5), were primarily detected in developing haploid spermatids [4,5]. These molecules are localized in testicular Leydig cells, epididymal spermatozoa and semen [4,5,6]. Interestingly, many initial components of sweet/umami taste signaling cascades, such as PLCβ2, G protein γ13 subunit (Gγ13), and transient receptor potential channel M5 (TRPM5), are also expressed in testicular Leydig cells and late spermatogenic cells [7,8]. In addition, T1R1/T1R3 expressed in the flagella and acrosomal cap of sperm in mice regulates basal Ca^2+^ and cAMP levels during sperm development, maturation, and fertilization [6,9]. Genetic absence of both *Tas1r3* and G protein subunit alpha transducin 3 (*Gnat3*) leads to specific sterility in male mice [10]. Clofibrate-mediated inhibition of humanized *TAS1R3* in *Tas1r3*−/−, *Gnat3*−/− double-null mice leads to inducible male sterility [10]. In our previous studies, we have shown that mouse testicular T1R3 and its associated heterotrimeric Gαgust are expressed in the testis in a stage-dependent manner during development, and with a cell-specific pattern during the spermatogenic cycle [11].

Owing to the role of T1R3/Gαgust in spermatogenesis and steroidogenesis in the mouse testis [9,11,12], we hypothesized that *TAS1R3* contributes to testis development in the Congjiang Xiang pig, a rare Chinese indigenous breed characterized by a small body size, with an adult body weight of approximately 40 kg (while other minipigs are about 60 kg) [13]. They reach sexual maturity at the age of around 3−4 months and have high disease resistance, favorable meat quality and strong adaptability [13,14,15]. Since Congjiang Xiang pigs share many anatomical and physiological features with humans (including the organ weights and blood physiological and biochemical indicators) [13] and their relatively small size makes them easier to handle and more manageable than other varieties of pigs [13], they are a potential model for biomedical research and xenotransplantation [16]. Studies of Congjiang Xiang pigs have focused on the determination of their characteristics as germplasm resources [14,15], and little is known about the gene regulatory mechanisms underlying the highly complex cellular remodeling associated with spermatogenesis and male hormone secretion. In the present study, we examined the expression profiles of T1R3 in the testes of Congjiang Xiang pigs; our results provide insight into the process of testis development and the onset of the first spermatogenic wave in the breed.

## 2. Material and Methods

### 2.1. Animals and Ethics Statement

All animal procedures were approved by the Institutional Animal Care and Use Committee of Guizhou Medical University (No. 1801227, Guiyang, China) and the Guizhou University Experimental Animal Ethics (No. EAE-GZU-2020-P001, Guiyang, China). All experimental procedures were conducted in accordance with the Regulations for the Administration of Affairs Concerning Experimental Animals approved by the State Council of the People’s Republic of China. Male Congjiang Xiang pigs were obtained from the Guizhou Lushengyuan Animal Husbandry Technology Development Co., Ltd. (Guiyang, China) at postnatal day 15, 30, 60, 90, 120, and 180. Each time-point had six individual animals. Newborn piglets were housed with their mother sows before weaning and each lactating sow was housed in an individual pen. Eighteen piglets were castrated at 15, 30, and 60 day, respectively. The uncastrated piglets (*n* = 18) were weaned at 60 day and then also housed in individual pens with ad libitum access to water and a well-ventilated room maintained at 20−26 °C and 50−70% humidity until sampling. They were fed 3 meals per day at volumes to ensure free feed intake.

### 2.2. Experimental Design

Based on the testis stereology and morphology, as well as the plasma testosterone concentrations of Congjiang Xiang pigs at six time-points during postnatal development (i.e., 15 day, 30 day, 60 day, 90 day, 120 day, and 180 day), key stages (including prepuberty: 15 day, 30 day; early puberty: 60 day; late puberty: 90 day; sexual maturity: 120 day, 180 day) related to testis development were identified. The expression levels of T1R3 and the associated signaling pathway components T1R1 and PLCβ2 were evaluated at the above-mentioned stages of testis development by Western blotting (WB), immunohistochemical (IHC) analysis and quantitative real-time PCR (q-PCR). In addition, the expression patterns of T1R3 and PLCβ2 during the spermatogenic cycle of Congjiang Xiang pigs at sexual maturity were determined by IHC.

### 2.3. Tissue Preparation

Prior to sampling, the body weight of each animal (age 15–180 day) was recorded. Blood (from the jugular vein, 5 mL) and testes were collected after animals were anesthetized by 0.04 mg/kg atropine sulfate salt monohydrate (A0257; Sigma-Aldrich. St. Louis, MO, USA) via intramuscular injection in the hip. Surgical castration involved making an incision over the disinfected scrotum via the vaginal tunic and removing the testis by a combination of traction and twisting. The wound was treated with antibiotics and an antiseptic spray and was left open to heal. All surgical procedures were performed by a veterinarian. The plasma was separated from blood by centrifugation at 4000 rpm for 10 min and stored at −80 °C for hormone evaluation. After orchiectomy, the two sides of the testes in each boar were weighed and harvested immediately. Testis samples were taken from the central region of each of the left testes and washed three times with phosphate-buffered saline (PBS). Next, each sample was divided into two parts, and immediately frozen in liquid nitrogen and stored at −80 °C for RNA preparation and WB, respectively. Each of the right testes were removed and cut into 2 portions of 1 cm^3^ (10 nm × 10 nm × 10 nm) in volume. According to our previous studies [17], one portion was fixed in modified Davidson’s fluid (mDF) for hematoxylin and eosin (H&E) staining and the other portion was fixed in 4% paraformaldehyde (PFA) for IHC analysis. Additionally, 60 day mouse testis sections were donated by Prof. Fangxiong Shi’s group (Nanjing Agricultural University, Nanjing, China).

### 2.4. Testosterone Assay

Plasma hormone concentrations of testosterone were determined by an enzyme-linked immunosorbent assay (ELISA) using a commercial Porcine Testosterone (T) Elisa Kit (201902; Nanjing Jiancheng Bioengineering Institute, Nanjing, China). Detection was performed at 450 nm using a Varioskan LUX Multimode Microplate Reader (Thermo Fisher Scientific, Waltham, MA, USA). The lower limit of detection of the kit was 0.6 nmol/L, and the intra- and interassay coefficients of variation were <10% and 12%, respectively. Assays were performed in triplicate.

### 2.5. Quantitative Real-Time PCR (q-PCR)

Three animal samples from each time-point passed quality control, and were selected for q-PCR assay.

Total RNA was extracted from porcine testes at prepuberty (30 day), early puberty (60 day), late puberty (90 day), and sexual maturity (120 day) using TRIzol Reagent (15596026; Thermo Fisher Scientific, Inc., Waltham, MA, USA) according to the manufacturer′s protocol. Total RNA was dissolved in 30 µL RNase-free deionized water and stored at −80 °C. The quality of RNA was assessed by a NanoDrop 2000 spectrophotometer (Thermo Fisher Scientific, Wilmington, DE, USA), and the RNA samples had A260/280 ratios of approximately 1.98−2.14, A260/230 ratios ranged between 2.01−2.20, and high integrity (Figure S1 and Table S1), which is acceptable for downstream applications [18]. For each available sample (Table S1), cDNA was synthesized using the Revert Aid First Strand cDNA Synthesis Kit (K1622; Thermo Fisher Scientific, Inc., Waltham, MA, USA) and used as a template for PCR.

q-PCR was performed on the CFX96 real-time PCR system (Bio-Rad, Hercules, CA, USA) using Power UP SYBR GREEN Master Mix (A25742, Thermo Fisher Scientific, Inc., Waltham, MA, USA).

The PCR amplification was performed in a total volume of 10 µL containing 5 µL of 2 × Es Taq Master Mix, 2 µL of RNase-free dH_2_O, 1 µL of cDNA, and 1 µL each of forward and reverse primers (10 pmol/µL). Each PCR cycle included a denaturation step at 95 °C for 3 min, followed by 40 cycles of 95 °C for 15 s, 58.4−60 °C for 15 s, and extension at 72 °C for 1 min, and a dissociation step consisting of 95 °C for 15 s, 60 °C for 1min, and 95 °C for 15 s. Gene expression levels of target genes (*TAS1R1*, *TAS1R3*, and *PLCB2*) were normalized against levels of the selected housekeeping gene *ATCB* [19] (Figure S2 and Table S2) and expressed as 2^−ΔΔCT^ [20] using samples on day 30 for calibration. The standard curve for each primer pair was run in one plate for q-PCR. The primers for q-PCR are listed in Table 1. The amplification efficiency of all primer pairs ranged from 90.1–102.0%, and the R^2^ values were greater than 0.99 (Figure S3).

### 2.6. Histological Examination

Testis samples were fixed in mDF at 4 °C for 24 h for histological experiments [17]. The fixed testes were trimmed, subjected to routine histologic processing, and paraffin-embedded. The well-fixed paraffin-embedded testis sections were cut at 5 μm using a microtome (RM2235; Leica Biosystems Nussloch Gmbh, Nussloch, Germany), mounted on slides coated with 3-aminopropyl-triethoxysilane (APES, 440140; Sigma-Aldrich, St. Louis, MO, USA) and dried for 24 h at 37 °C. Subsequently, the sections were dewaxed with xylene, hydrated by a gradient ethanol series, and washed in ddH_2_O three times (5 min/each). Next, they were stained with hematoxylin and eosin (H&E, G1120; Solarbio Life Sciences, Beijing, China) according to the manufacturer′s protocol. After embedding in neutral resin, the stained sections were viewed under a Nikon C2 Confocal Microscope (Tokyo, Japan). Morphological changes of the testis during postnatal development were evaluated using 12 slices (2 slices/testis × 2 testes × 3 animals) at each stage of development.

### 2.7. Immunohistochemistry

To examine the localization of T1R3, T1R1, gustducin α-3 chain (GNAT3) and PLCβ2 in testes from the Congjiang Xiang pigs at prepuberty (30 day), early puberty (60 day), late puberty (90 day), and sexual maturity (120 day), IHC analyses were performed with the streptavidin-biotin complex (SABC) method [21]. Testis samples were fixed in 4% PFA at 4 °C for 24 h and processed in a series of graded ethanol solutions [17]. Testicular sections were deparaffinized and hydrated via graded xylene and ethanol, followed by heat-induced antigen retrieval in 0.01 M citrate buffer (pH, 6.0; microwave oven, 100 °C, 5 min). To avoid endogenous peroxidase activity and nonspecific antibody staining, the sections were blocked with 3% H_2_O_2_ in methanol and 5% bovine serum albumin (BSA, A4737; Sigma-Aldrich, St. Louis, MO, USA) for 1 h at room temperature. Antibodies for T1R3, T1R1, GNAT3, and PLCβ2 were diluted in PBS (P1020; Solarbio Life Sciences, Beijing, China) containing 1% BSA, and the sections were incubated overnight at 4 °C with primary antibodies (Table 2). A Rabbit IgG-SABC Kit (SA2002; Boster Biological Technology, Wuhan, China) was used to detect the immunoreactivity of these proteins. The immunoreactivity was visualized with 3,3′-diaminobenzidine tetrahydrochloride (DAB) enhanced liquid substrate system (D3939, Sigma-Aldrich, St. Louis, MO, USA) according to the manufacturer′s protocol. The negative control sections were incubated with PBS instead of the primary antibody. Finally, the reacted sections were counterstained with hematoxylin solution (G1120; Solarbio Life Sciences, Beijing, China) for 50 s, mounted with coverslips and viewed under a Nikon C2 Confocal Microscope (Tokyo, Japan). Antibodies applied in IHC are listed in Table 2.

Antibody staining intensities were determined by three independent observers, following a previously described method [22]. Immunostaining was evaluated using 18 slices (3 slices/testis × 2 testes × 3 animals) for each protein at each stage of development. 

### 2.8. Sodium Dodecyl Sulfate Polyacrylamide Gel Electrophoresis (SDS-PAGE) and WB

Three animal samples from each time-point passed quality control, and were selected for WB assay. The protein extracts from porcine testes (80 mg) were collected in radioimmunoprecipitation assay (RIPA) buffer (P0013B; Beyotime, Nantong, China) containing 10 mM phenylmethyl-sulfonyl fluoride (PMSF, ST506; Beyotime, Nantong, China) using a FastPrep-24^TM^ Classic bead beating grinder and lysis system (MP Biomedicals, Santa Ana, CA, USA). The supernatant was separated and the protein concentration was determined using a Bicinchoninic Acid Protein Assay Kit (PC0020; Solarbio Life Sciences, Beijing, China). Twelve sample lysates (30 µg, 15 µL) and a protein standard ladder (P0068; Beyotime, Nantong, China) were separated on a 10% (*w/v*) SDS-PAGE gel (2 h, 100 V; P1200; Solarbio Life Sciences, Beijing, China), and then transferred (1.5 h, 100 V) onto a polyvinylidene fluoride membrane (K5AA6843H; Millipore, Bedford, MA, USA).

After hydrating in methanol and deionized ddH_2_O, the membrane was blocked with 5% skimmed milk in Tris-buffered saline buffer with Tween 20 (TBST, 20 mM Tris-buffered saline, 0.05% Tween 20, pH 7.5) for 2 h at room temperature, incubated with diluted T1R3 antibody (ab150525; Abcam, MA, USA) for 16 h at 4 °C, rinsed three times in TBST, incubated with horseradish peroxidase-linked secondary goat anti-rabbit IgG (AC026; ABclonal Technology, Wuhan, China) at room temperature for 2 h, and then rinsed four times with TBST. All blocks, incubations, and rinses were performed on a rocking platform. Subsequently, protein bands on one blot were detected with Pierce^TM^ ECL Western Blotting Substrate (400 µL, 1 min; 32109; Thermo Fisher Scientific, Inc., Waltham, MA, USA) and visualized using the Molecular Imager ChemiDoc XPS+ system (Bio-Rad Laboratories Co., Ltd., CA, USA). To control for loading efficiency, the blots were stripped with Western Blot Stripping Buffer (ab270550; Abcam, MA, USA) at room temperature for 20 min and re-probed with β-actin antibody (AF7018; Affinity Biosciences, VIC, Australia). The T1R3 protein bands were normalized to β-actin. The intensities of immunoreactive bands were evaluated using the ImageJ (http://rsbweb.nih.gov/ij/ (accessed on 29 January 2021)) to quantify the target protein levels by densitometry. Antibodies applied in WB are listed in Table 2.

### 2.9. Statistical Analysis

Results are expressed as means ± standard deviation (SD). Statistical analyses were performed using GraphPad Prism 8.0.1 (GraphPad Software, Inc., La Jolla, CA, USA). Data were analyzed by one-way analysis of variance (ANOVA) with Tukey’s range tests for multiple comparisons between groups. A significant difference was defined as *p* < 0.05.

## 3. Results

### 3.1. Changes in the Gonadosomatic Index and Testosterone Level during Development

**Figure 1:**
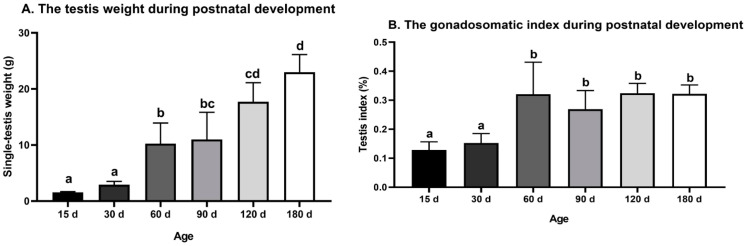
Testis weight and gonadosomatic index during postnatal development of the Congjiang Xiang pig. (**A**) The testis weight of Congjiang Xiang pigs from 15 day to 180 day. (**B**) The gonadosomatic index of Congjiang Xiang pigs from 15 day to 180 day was calculated by dividing testis weight by body weight. Measurements are means of left and right testes of a pair. All results are indicated by means ± SD (*n* ≥ 3). The data was analyzed by one-way ANOVA with Tukey’s multiple comparisons. Values not sharing the same letter (a–d) denote significant differences among groups (*p* < 0.05).

**Figure 2:**
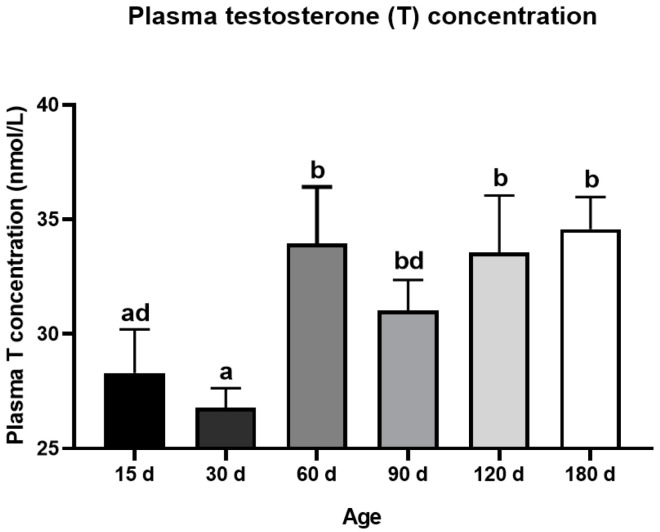
Dynamic changes of plasma testosterone level during postnatal development of the Congjiang Xiang pig. The blood was collected from the jugular vein by a board-certified veterinarian. Results are indicated by means ± SD (*n* ≥ 3). The data was analyzed by one-way ANOVA with Tukey’s multiple comparisons. Values not sharing the same letter (a,b,d) denote significant differences among groups (*p* < 0.05).

The testis weight of the Congjiang Xiang pig (Figure 1A) increased from 15 to 180 day (*p* < 0.0001). The testis index increased from 15 to 60 day (*p* = 0.001), with no significant changes thereafter (*p* > 0.05), as shown in Figure 1B. The accelerated increase in the testis weight and testis index at postnatal 60 day corresponded with higher levels of plasma testosterone than those at 15 day *(p* = 0.0007) and 30 day (*p* < 0.0001); plasma testosterone mainly acts via androgen receptors on Sertoli cells to maintain normal testicular function (Figure 2). After completing the first wave of spermatogenesis at 60 day, the testis index and testosterone level showed no changes with age (*p* > 0.05), regardless of the increased testis weight from 60 day to 180 day (*p* < 0.0001), as shown in Figure 2.

### 3.2. Morphological Changes in the Testes during Development

**Figure 3:**
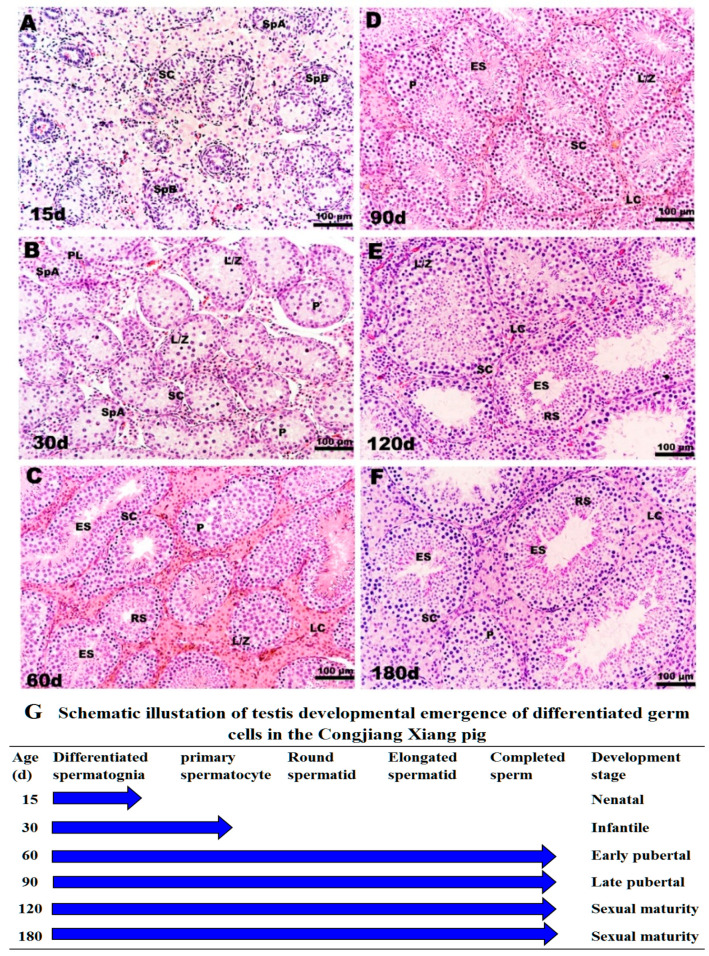
Histological observation of testes in Congjiang Xiang pig during development: (**A**–**F**) represent 15, 30, 60, 90, 120 and 180-day-old the Congjiang Xiang pig testes (*n* = 3). (**G**) A schematic illustration of testis development emergence of differentiated germ cells in the Congjiang Xiang pig according to Yomogida K. (2009) [23]. Testes were removed and fixed in mDF 24 h, embedded in paraffin, sectioned (5 μm) and stained with Hematoxylin and Eosin using standard procedures. Leydig cells (LC); Sertoli cells (SC); spermatogonia types A (SpA); spermatogonia types B (SpB); leptotene and zygotene spermatocytes (L/Z); preleptotene spermatocytes (PL); pachytene spermatocytes (P); round spermatids (RS); elongating/elongated spermatids (ES). Scale bars (black line in the bottom-right of pictures): A–F = 100 µm (200 × magnification).

The specific morphological and functional features during testicular ontogeny from 15 day to 180 day are displayed in Figure 3. Consistent with testis weight data, 15-day testes consisting of tubules were lined by spermatogonia types A and B and mitotically active Sertoli cells; the regressing fetal Leydig cells (FLC) were replaced with progenitor Leydig cells (PLC) in a large area of the interstitium (Figure 3A). From 15 day to 30 day, Sertoli cells stopped dividing and spermatocyte development ensued. The lumen was visible in a few tubules with preleptotene, leptotene, zygotene and pachytene spermatocytes, as shown in Figure 3B. The interstitium contained PLC and a small number of immature Leydig cells, which stimulate meiosis. There were no clear morphological differences between FLC, PLC, and immature Leydig cells. After 30 day, pachytene spermatocytes, round spermatids and elongating or elongated spermatids were present in the tubules, and tubule diameters increased at 60 day (Figure 3C). After the first wave of spermatogenesis was complete, the tubular diameter, cell number, and density of elongating spermatids increased gradually from 90 day to 180 day, consistent with the observed changes in testis weight (Figure 3D–F).

According to morphological features, testes weights, and plasma testosterones levels, four development stages were identified in male Congjiang Xiang pigs as follows (Figure 3G): pre-pubertal (15 day, 30 day), early pubertal (60 day), late pubertal (90 day), and sexual maturity (120 day, 180 day).

### 3.3. Expression of T1R3 and PLCβ2 in Testes of Congjiang Xiang Pigs during Postnatal Development

**Figure 4:**
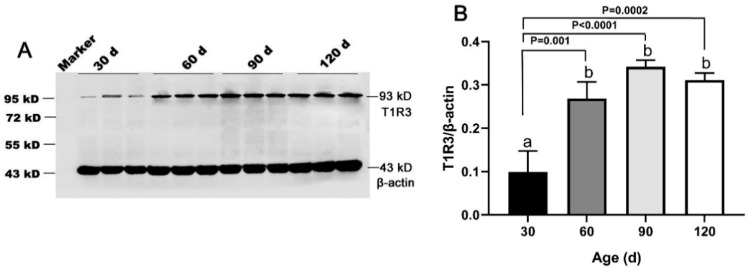
Expression of T1R3 in testes of Congjiang Xiang pigs at four ages. Tissues were collected from pigs at 30, 60, 90, and 120 days old. (**A**), the protein expression of T1R3 in testes from 30–120 day with western blot. (**B**), the relative expression of T1R3 from 30 to 120 day analyzed by the Image J. The expression pattern of T1R3 was measured by WB, β-actin was used as an internal control. The relative expression level was calculated by dividing the T1R3 signal intensity by corresponding β-actin intensity. Results are indicated by means ± SD (*n* = 3). Bars with different letters are significantly different (*p* < 0.05).

The T1R3 and β-actin protein bands were detected at 93 kD and 43 kD in both porcine testes and positive control (Figure S6), indicating the antibodies specificity. Our results showed that the expression of T1R3 increased approximately fivefold from 30 day to 60 day (*p* < 0.05) and remained at a high level until 120 day (Figure 4A,B).

**Figure 5:**
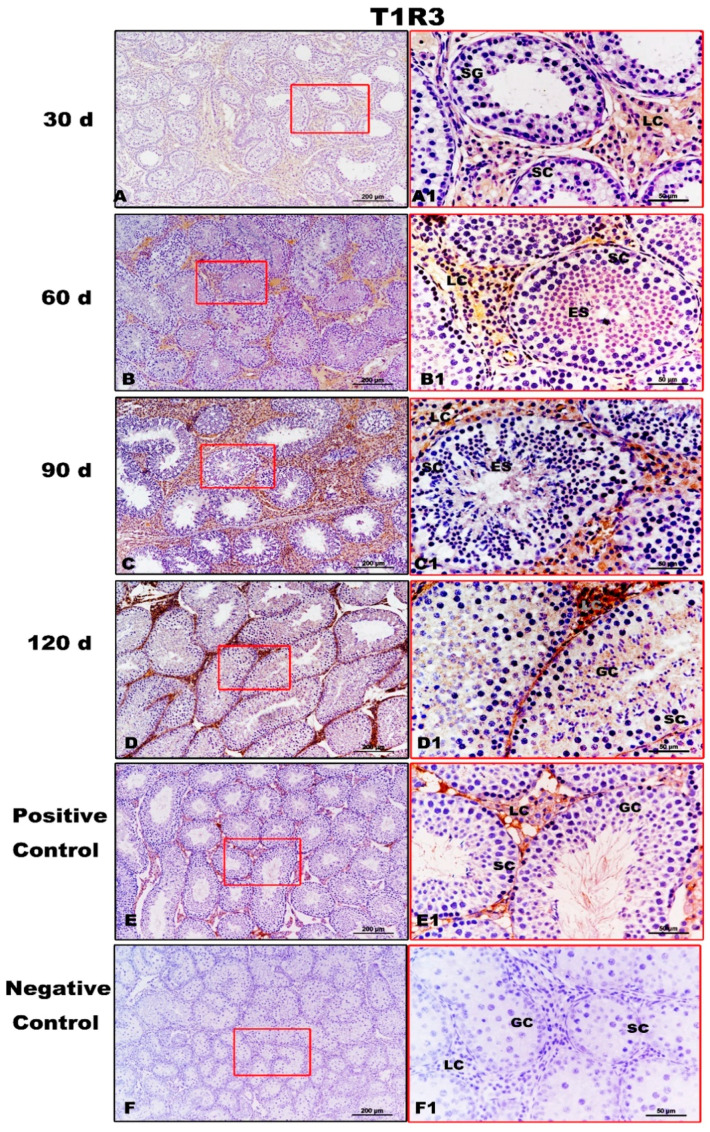
Immunolocalization of T1R3 protein in testes of Congjiang Xiang pigs during development. (**A**–**F**) represent the testis sections immunostained with primary antibody of T1R3 in 30-day-old Congjiang Xiang pigs, 60-day-old Congjiang Xiang pigs, 90-day-old Congjiang Xiang pigs, 120-day-old Congjiang Xiang pigs, positive control, and negative control groups (*n* ≥ 3/group), respectively. Picture (**E**), the mouse testis sections (at 60 day) were used as a positive control for T1R3. Picture (**F**), negative control, was performed with PBS instead of the primary antiserum of T1R3 (30 day Congjiang Xiang pig testes). Leydig cells (LC); Sertoli cells (SC); germ cells (GC); spermatogonia (SG); spermatocytes (P); elongating/elongated spermatids (ES). Pictures (**A**–**F**) marked by square boxes of red color, the boxes are enlarged beside and marked as (**A1**–**F1**). Scale bars: A1–F1 = 200 µm (100× magnification), A–F = 50 µm (400× magnification).

**Figure 6:**
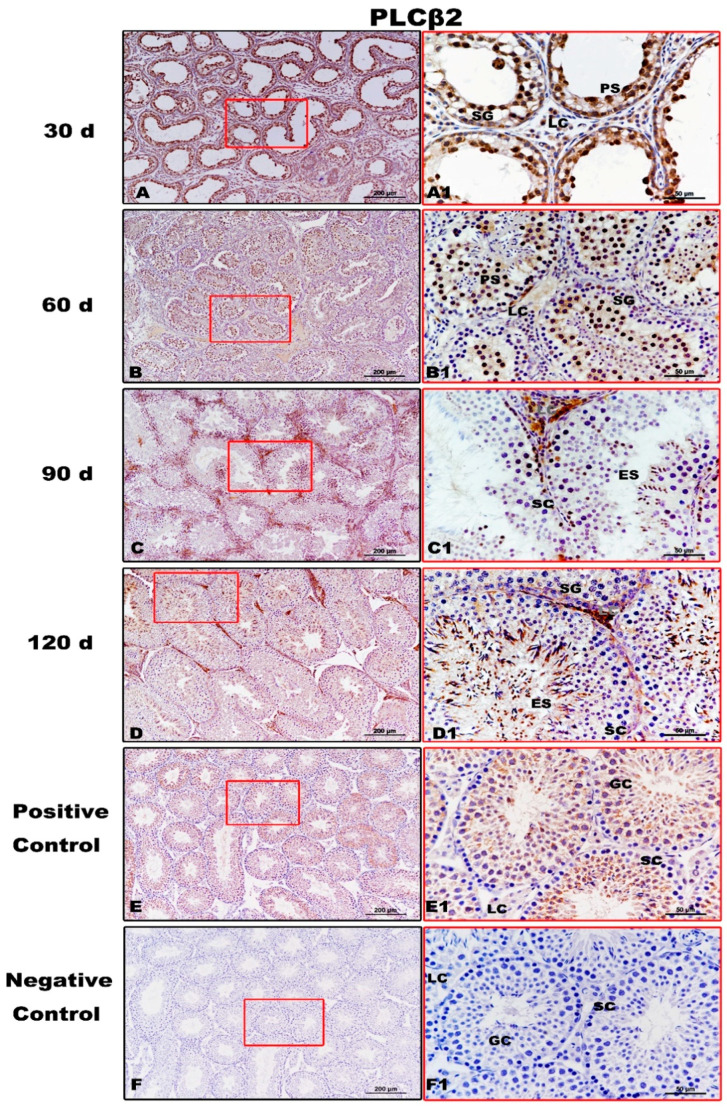
Immunolocalization of PLCβ2 protein in testes of Congjiang Xiang pigs during development. (**A**–**F**) represent the testis sections immunostained with primary antibody of PLCβ2 in 30-day-old Congjiang Xiang pigs, 60-day-old Congjiang Xiang pigs, 90-day-old Congjiang Xiang pigs, 120-day-old Congjiang Xiang pigs, positive control, and negative control groups (*n* ≥ 3/group), respectively. Picture (**E**) the mouse testis sections (at 60 day) were used as a positive control for PLCβ2. Picture (**F**) negative control, was performed with PBS instead of the primary antiserum of PLCβ2 (60 day mouse testes). Leydig cells (LC); Sertoli cells (SC); germ cells (GC); spermatogonia (SG); spermatocytes (P); elongating/elongated spermatids (ES). Pictures (**A1**–**F1**) are marked by square boxes of red color which are enlarged beside and marked as (**A1**–**F1**). Scale bars: A–F = 200 µm (100× magnification), A1–F1 = 50 µm (400× magnification).

T1R3 expression was strong in the testes of Congjiang Xiang pigs, as was the expression of the downstream taste signaling molecule PLCβ2, when the first spermatogenesis was completed after 60 day (Figure 5 and Figure 6). In addition, Leydig cells expressed the two proteins from 30 day to 120 day. At 30 day, T1R3 protein expression was detected in the Leydig cells rather than in the spermatogonia or Sertoli cells (Figure 5A,A1). However, positive signals for T1R3 were first recorded in the abluminal compartment of 60-day testes, where differentiating spermatids (elongating/elongated spermatids) were located (Figure 5B,B1). After puberty (90 day and 120 day), T1R3 immunoreactivity was recorded during spermatogenesis (Figure 5C,D,C1,D1). Testicular positivity for T1R3 was not detected when slides were incubated with the secondary antibody with the omission of primary antiserum (Figure 5F,F1).

PLCβ2, downstream of T1R3, showed evident immunolabeling in the spermatogonia, pachytene spermatocytes, and Leydig cells of 30-day testes (Figure 6A,A1). At 60 day, immunopositivity for PLCβ2 was detected from spermatogonia to elongating/elongated spermatids (Figure 6B,B1). From 90 day to 120 day, PLCβ2 immunoreaction was clearly observed during spermatogenesis (Figure 6C,D,C1,D1). No specific staining was detected in the negative control (Figure 6F,F1).

In summary, T1R3 and PLCβ2 immunolabeling increased gradually from 30 day to 60 day and then remained constant until 120 day. However, commercially available GNAT3 antisera and T1R1 antisera did not yield specific immunolabeling on mouse and pig testis tissues (Figures S4 and S5).

### 3.4. Expression of T1R3 and PLCβ2 during the Spermatogenic Cycle

**Figure 7:**
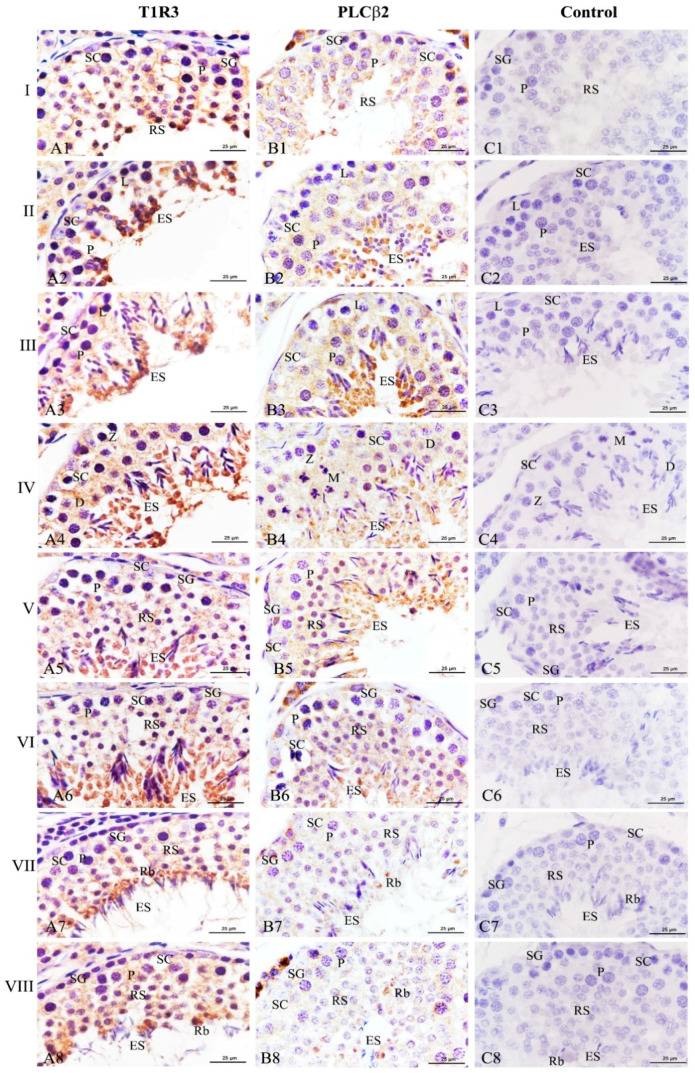
Expression of T1R3 and PLCβ2 during the spermatogenic cycle at 120 days in the testes of the Congjiang Xiang pig. Positive immunostaining in brown, and counterstaining with hematoxylin (*n* = 3). (**A1**–**A8**) represent immunostaining of T1R3 antibodies at stages I–VIII. (**B1**–**B8**) represent immunostaining of PLCβ2 antibodies at stages I–VIII. (**C1**–**C8**) represent negative control at stages I–VIII, which was performed with PBS instead of primary antibody. Leydig cells (LC); Sertoli cells (SC); spermatogonia (SG); leptotene and zygotene spermatocytes (L/Z); preleptotene spermatocytes (PL); pachytene spermatocytes (P); round spermatids (RS); elongating/elongated spermatids (ES); residual bodies (Rb). Scale bars: A1–A8 = 25 µm (1000× magnification); B1–B8 = 25 µm (1000× magnification); C1–C8 = 25 µm (1000× magnification).

To investigate whether T1R3 and PLCβ2 affected the spermatogenic cycle in Congjiang Xiang pigs, we analyzed their expression patterns in 120 day testes during the 8th of the spermatogenic cycle [21]. Throughout the spermatogenic cycle, the expression levels of T1R3 and PLCβ2 on the spermatogenic epithelium at stages II–VI, were higher than those at stages I, VII, and VIII (Figure 7). When round spermatids at stage I transformed into elongating/elongated spermatids at stages II–VI, T1R3 was significantly expressed in the cytoplasm of elongating/elongated spermatids, and the immunoreactions in these cells were higher than those in spermatogonia, leptotene/zygotene/pachytene/diplotene spermatocytes, and Sertoli cells at stages I–VI (Figure 7A1–A6). When the elongated spermatids were released at the luminal portion of the seminiferous tubule, residual bodies with strong T1R3 expression were observed at stages VII–VIII, and T1R3 was also detected at the flagella of elongated spermatids (Figure 7A7,A8).

Simultaneously, PLCβ2 exhibited a similar change from stages I–VI (Figure 7B1–B6). Although a significant decrease of this protein expression was found at stages VII–VIII, it is remarkable that PLCβ2 was only localized at the residual bodies (Figure 7B7,B8). No specific staining was detected in the negative control (Figure 7C,C1).

### 3.5. mRNA Expression of Umami Taste-Related Molecules in Testes of Congjiang Xiang Pigs during Development

**Figure 8:**
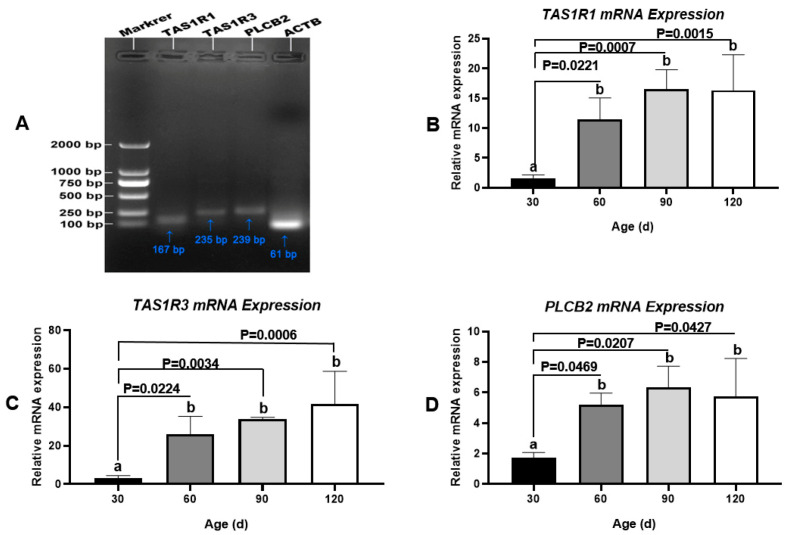
Transcription changes of umami tasting molecules, *TAS1R1*, *TAS1R3* and *PLCB2* during testis development of the Congjiang Xiang pig, evidenced by q-PCR. Gel electrophoresis q-PCR result of *TAS1R1* (167 bp), *TA1SR3* (235 bp), and *PLCB2* (239 bp), respectively. The mRNA levels of target genes were normalized with housekeeping gene (*ATCB,* 61 bp) and expressed as 2^−ΔΔCT^ using samples on day 30 as calibrator. Results are indicated by means ± SD (*n* = 3). Values not sharing the same letter (a, b) denote significant differences among groups (*p* < 0.05).

As an umami/amino acid receptor, T1R3 combines with T1R1 to regulate Ca^2+^ signaling via PLCβ2. These three proteins are encoded by *TAS1R1*, *TAS1R3*, and *PLCB2*, respectively (Figure 8A). As determined by q-PCR, the mRNA levels of these three genes were low in the testis at 30 day (pre-pubertal stage), increased substantially from 30 day to 60 day (early pubertal stage), and then remained steady until 120 day (sexual maturity) (Figure 8B–D). These findings agreed with the results of protein-level analyses. In addition, we did not detect the transcription of *TAS1R2* or *GNAT3* in Congjiang Xiang pig testes (Figure S7).

## 4. Discussion

In mammals, T1R3 plays a key role in sweet or umami perception along with T1R2 or T1R1, respectively, and transduces a common signaling cascade, G_gust_-PLCβ2-TRMP5-Ca^2+^, for the evaluation of foods [24]. Recent studies have demonstrated that this “tasting” locus is widely expressed from the mouse tongue to the testis; however, the precise roles of the taste receptor in the testis are unclear [8]. To elucidate the expression pattern of testicular T1R3 during testis development in the Congjiang Xiang pig, four key development stages were identified (pre-puberty, 30 day; early puberty, 60 day; late puberty, 90 day; sexual maturity, 120 day) in the present study, according to testicular parameters and testosterone levels [25]. The four developmental time-points were earlier than those of micro-minipigs (pre-pubertal 45 day; pubertal 90 day; and sexually mature 135 day) [26].

The expression of T1R3 in the porcine testes increased significantly from 30 day (prepuberty) to 60 day (early puberty), and remained high until 120 day (sexual maturity) along with PLCβ2, and only the expression levels of T1R1 at mRNA level were similar to those of T1R3. An IHC analysis showed that the strong expressions of testicular T1R3/PLCβ2 were detected at the cytoplasm of elongating/elongated spermatids and Leydig cells. The observed T1R3 expression pattern was consistent with the results of our previous study of mice [11]. The expression pattern of T1R3 and its related taste signaling components in germ cells and their potential functions have mainly been reported in mice [6,9,10,12,27,28]. The components of sweet/umami taste-related signaling cascades, such as T1R1, T1R2, T1R3, G_αgust_, G_γ13gust_, PLCβ2, and TRMP5, have been detected in spermatogenic cells of mice, in some cases, these molecules have been observed in the testicular basal layer, Leydig cells and epididymal spermatozoa [7,8]. Using a double-knockout mouse line, Mosinger et al. (2013) showed that the absence of both T1R3 and G_αgust_ leads to male-specific sterility [10]. The double-null mice display exfoliated spermatids and numerous giants and pyknotic cells in the testicular tubule lumen as well as immature cells in the epididymis and nonmotile sperm, which are related to impaired cAMP-regulated gene transcription (CREM) [10]. These data indicate that T1R3 and G_αgust_ are functionally crucial in spermiogenesis, sperm development, and maturation. It is likely that mouse T1R3 acts in concert with T1R1, as *Tas1r1* deficient mice show spermatogenic abnormalities (e.g., an increase in multinucleated giant cells and miss-located spermatocytes) and increased apoptosis during spermatogenesis [6,9]. The *Tas1r1* null-mutant sperm show increased spontaneous acrosome reactions as well as increased intracellular Ca^2+^ and cAMP levels [6]. Therefore, it was remarkable that apart from T1R3, the umami/sweet taste-related signaling component PLCβ2 was detected in porcine testes, in addition to the mRNA expression of T1R1. These data provide evidence that T1R3 could contribute to the signaling in the mammalian testis.

Interestingly, increases in testicular T1R3, T1R1, and PLCβ2 levels are in accordance with significant increases in plasma testosterone and the testis index from 30 day to 60 day. In the testis, the changes in testosterone are related to the fate of Leydig cells from the postnatal period to puberty. In mice, a four-stage model of Leydig cell development has been identified and characterized as fetal to progenitor to immature to mature Leydig cells, and only fetal and immature Leydig cells produce testosterone [29,30]. Similarly, there was significant testicular steroidogenic activity in newborn male pigs, and testicular steroid levels declined dramatically at the prepubertal stage (2 to 5 months) [31]. When Leydig cells reach final maturation, the secretion of testosterone is equal to that in the adult stage [31]. Apart from seminiferous tubules, T1R3 and PLCβ2 were mainly localized in testicular Leydig cells of the Congjiang Xiang pig; however, the role of these proteins in Leydig cell development remains unknown. We have previously shown that mouse testis weights and testosterone levels increase with the up-regulation of key molecules involved in testicular sweet/umami taste (T1R3 and G_α-gust_) and steroidogenesis (steroidogenic acute regulatory protein, StAR, etc.) after administration of saccharin sodium in mouse food and drink [12]. Steroidogenesis is essential for normal testis development and function. In Leydig cells, luteinizing hormone (LH) secreted by the pituitary binds to the LH receptor (LHCGR) to induce a LHCGR-complex interaction, which activates the cAMP cascade to initiate a classical steroidogenic pathway [32,33,34]. Therefore, we speculate that in addition to increased testosterone in the plasma, high levels of T1R3 and PLCβ2 in the testis after puberty may be related to the regulation of steroidogenesis in Leydig cells via the T1R3-PLCβ2 pathway. A better understanding of gene regulation in testicular Leydig cells development and spermiogenesis may provide a basis for limiting the incidence of male reproductive pathologies caused by the dysfunction of Leydig cells [35].

In addition, the localization of T1R3 and PLCβ2 in the cytoplasm of elongating or elongated spermatids in Congjiang Xiang pigs as well as residual bodies was consistent with previous results for mice [11]. However, the role of T1R3 coupled with T1R1 and PLCβ2 in residual bodies needs to be clarified in the future.

The minipig is considered a convenient animal model for preclinical studies because they rapidly achieve sexual maturity (before the age of six months) and the duration of the spermatogenic cycle is short (i.e., 35 day) [36]. Although there are structural variations at the genome level between relatively large litter sizes (average litter size: 13 piglets) and small litter sizes (average litter size: 8 piglets) in Congjiang Xiang pigs for long-term grazing and inbreeding [37,38], the breed is clearly characterized by early sexual maturity [39,40]. According to our study, Congjiang Xiang pigs exhibit the onset of puberty at approximately 2 months [40], similar to Gottingen pigs, and earlier than Yucatan minipigs (3.6–4 months) and Yorkshire (around 6 months) [41]. The testes of Yorkshire pigs are remarkably active in steroidogenesis with a peak at 14–28 days (about 5–10 nmol/L) after birth, remain at low activity from 60 to 150 day (2–5 nmol/L) and increase steroidogenic activity thereafter (6 months: 10–20 nmol/L) [42]. During testis development, serum testosterone levels increase sharply at the prepubertal to pubertal phase in male mammals [43]. In the present study, the plasma testosterone levels were also low in the prepubertal period of Congjiang Xiang boars, increased substantially between 30 day and 60 day old (about 26–37 nmol/L), and remained high thereafter (6 months: 32–36 nmol/L). In addition, the early age of the postnatal period is stable, despite a low trend of plasma testosterone from 15 day to 30 day, probably due to the regression of FLCs and the development of PLCs that dominantly produce dihydrotestosterone and 3α-androstanediol, rather than testosterone [29]. Low testosterone does not preclude spermatogonia development, and the increase in testis weight slows during this period. In summary, these data confirmed that the change in plasma testosterone levels was closely associated with testis development in Congjiang Xiang pigs.

## 5. Conclusions

Our results provide clear evidence for the expression of T1R1, T1R3, and PLCβ2 in the porcine testis. T1R3 and PLCβ2 were expressed in both tubular and interstitial compartments of the testis during postnatal development. The expression levels of T1R3 and PLCβ2 increased from 30 to 60 day and remained high until 120 day. During the spermatogenic cycle, the two proteins exhibited the stage- and cell-specific expression patterns; the expression levels at stages II–VI were higher than those at other stages, while late spermatids and residual bodies expressed much higher levels than those of other cells in the seminiferous epithelium. These data indicate a possible role of T1R3 in the regulation of spermatid differentiation and Leydig cell function. The precise roles of testicular T1R3 and the related taste signaling component PLCβ2 need to be clarified in the future.

## Figures and Tables

**Table 1:** The primers for q-PCR.

Gene Name	NCBI Reference Sequence	Oligonucleotide Primers (5′-3′)	Expected Products Size (bp)	Melting Temperature (Tm, °C)	Amplification Efficiency (%)
*TAS1R3*	NM_031872.2	Forward: TCATCACCTGGGTTTCCTT	235	60	90.1
		Reverse: GGGGTCATTTGTTTTTTCC			
*TAS1R1*	NC_010448.4	Forward: GGAGATCCGCAAGGTCAAT	167	58.4	101.6
		Reverse: GCTGAACTGGCGACAACAAT			
*PLCB2*	XM_021097762	Forward: CACCACCCTTTCTATTACGG	239	59	98.6
		Reverse: TGTTGCCTTCCTCCATCA			
*ACTB*	XM_021086047.1	Forward: AAGTACTCCGTGTGGATCGG	61	60	102.0
		Reverse: ACATCTGCTGGAAGGTGGAC			

**Table 2:** Antibodies were applied in immunohistochemistry (IHC) and Western blotting (WB).

Manufacturer	Protein/Name	Species Origin	Catalogue No.	Dilution *	
				WB	IHC
Affinity Biosciences	T1R1	Rabbit	DF10278	−	−
Abcam	T1R3	Rabbit	ab150525	1/1000	1/150
Thermo Fisher Scientific	GNAT3	Rabbit	PA5-50670	−	−
Thermo Fisher Scientific	PLCβ2	Rabbit	PA5-75551	−	1/200
Affinity Biosciences	β-actin	Rabbit	AF7018	1/3000	−
ABclonal Technology	Second antibody	Rabbit	AC026	1/5000	−
Boster Biological Technology	SABC Kit		SA2002	−	1:300

Note: * Tris-buffered saline buffer with Tween 20 (TBST) and phosphate-buffered saline (PBS) were used as the dilution of antibodies applied in WB and IHC, respectively.

## Data Availability

The data presented in this study are available on request from the corresponding author.

## Supplementary Materials

The following are available online at https://www.mdpi.com/2076-2615/11/2/437/s1. Figure S1: The quality of the total RNA samples extracted from Congjiang Xiang pig tests measured by RNA electrophoresis; Figure S2: Ct values of four candidate housekeeping genes across testis sample at 30–90 day; Figure S3: The amplification and standard curve of TAS1R3, TAS1R1, PLCB2 and ACTB in Q-PCR experiment; Figure S4: Expression of T1R1 and GNAT3 in testes of Congjiang Xiang pigs at 30, 60, 90 and 120 days old; Figure S5: Immunolocalization of T1R1 and GANT3 proteins in testes of Congjiang Xiang pigs and mice; Figure S6: WB experiment of T1R3 antibody in the Congjiang Xiang pig testes at 30–120 day; Figure S7: Transcription of TAS1R1, TAS1R2, GNAT3 and PLCB2 in the adult Congjiang Xiang pigs; Table S1: Yields and purity of total RNA measured with NanoDrop; Table S2: The Ct values of four housekeeping genes during developmental stages of porcine testis.
