# Supplementary material for: Longitudinal Expression of Testicular TAS1R3 from Prepuberty to Sexual Maturity in Congjiang Xiang Pigs
Source: Animals (Basel). 2021 Feb 8;11(2):437. doi: 10.3390/ani11020437 (PMC7916009; doi:10.3390/ani11020437)
Supplement: Supplementary file 1 [file animals-11-00437-s001.pdf]

animals

Supplementary materials

Longitudinal Expression of Testicular *TAS1R3* from Prepuberty to Sexual Maturity in Congjiang Xiang Pigs

Ting Gong [1,2,3], Weiyong Wang [1,2,3], Houqiang Xu [1,2,3,*], Yi Yang [1,2,3], Xiang Chen [1,2,3], Lijie Meng [1,2,3], Yongjian Xu [1,2,3], Ziqing Li [3], Sufang Wan [3] and Qi Mu [3]

[1] Key Laboratory of Animal Genetics, Breeding and Reproduction in The Plateau Mountainous Region, Ministry of Education, Guizhou University, Guiyang 550025, China; tgong@gzu.edu.cn (T.G.); wangxiaochui369@163.com (W.W.); yiyangnutrition@foxmail.com (Y.Y.); as.xchen2@gzu.edu.cn (X.C.); mlj18208544454@163.com (L.M.); xuyongjian0503@163.com (Y.X.)
[2] Key Laboratory of Animal Genetics, Breeding and Reproduction, Guiyang 550025, China
[3] College of Animal Science, Guizhou University, Guiyang 550025, China; ZQli98@163.com (Z.L.); w18385462413@163.com (S.W.); Muqi0524@163.com (Q.M.)
* Correspondence: houqiangxu2020@163.com; Tel: +0851-88298005

Citation: Gong, T.; Wang, W.; Xu, H.; Yang, Y.; Chen, X.; Meng, L.; Xu, Y.; Li, Z.; Wan, S.; Mu, Q. Longitudinal Expression of Testicular *TAS1R3* from Prepuberty to Sexual Maturity in Congjiang Xiang Pigs. *Animals* **2021**, *11*, 437. https://doi.org/10.3390/ani11020437

Academic Editor: Olli A.T. Peltoniemi

Received: 2 December 2020

Accepted: 29 January 2021

Published: 8 February 2021

Publisher's Note: MDPI stays neutral with regard to jurisdictional claims in published maps and institutional affiliations.

Copyright: © 2021 by the authors. Licensee MDPI, Basel, Switzerland. This article is an open access article distributed under the terms and conditions of the Creative Commons Attribution (CC BY) license (http://creativecommons.org/licenses/by/4.0/).

Supplementary materials

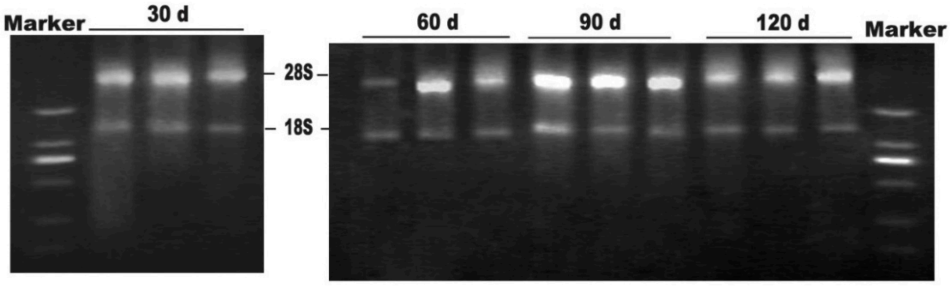

Figure S1. The quality of the total RNA samples extracted from Congjiang Xiang pig testes measured by RNA electrophoresis. According to the TAE/formamide method described by Maseka (2005) [1], the total RNA samples extracted from Congjiang Xiang pig testes (at 30 d, 60, 90 d and 120 d) were run in 1.2% agarose gels containing 1×TAE buffer (0.04 M Tris-acetate, 1 mM EDTA). DNA Marker: 2000 bp, 1000 bp, 750 bp, 500 bp, 250 bp, 100 bp. The 28S and 18S RNA bands are indicated, and the two clear rRNA bands detected by RNA electrophoresis suggests that the RNA is relatively intact.

Table S1. Yields and purity of total RNA measured with NanoDrop.

Age	Number	Concentration (μg/μL)	A260/280[1]	A260/230[2]	Availability
30 d	1	1.28	1.9	1.5	no
	2	2.56	2.02	1.73	no
	3	4.00	1.98	2.09	yes
	4	7.47	2.21	2.14	no
	5	5.27	2.03	2.01	yes
	6	6.99	2.11	2.19	yes
60 d	1	2.43	2.11	2.2	yes
	2	1.04	1.84	1.32	no
	3	1.74	1.97	1.92	no
	4	2.43	2.11	2.2	yes
	5	4.78	2.13	2.2	yes
	6	3.64	2.08	2.04	yes
90 d	1	2.18	2.14	2.11	yes
	2	2.27	2.03	1.82	no
	3	1.06	1.97	1.61	no
	4	4.65	2.11	1.98	no
	5	3.71	2.11	2.19	yes
	6	3.09	2.1	2.11	yes
120 d	1	3.9	2.07	2.26	no
	2	3.2	2.07	2.38	no
	3	4.53	2.09	2.20	yes
	4	2.18	2.14	2.11	yes
	5	1.18	2.09	2.03	yes
	6	2.44	2.05	1.79	no

Notes: [1] aratio of A260/280 around 2.0 is generally accepted as "pure" for RNA, <1.7 indicates a contaminated RNA; [2] a ration of A260/230 ranged from 2.0-2.2 is acceptable for q-PCR analysis [2].

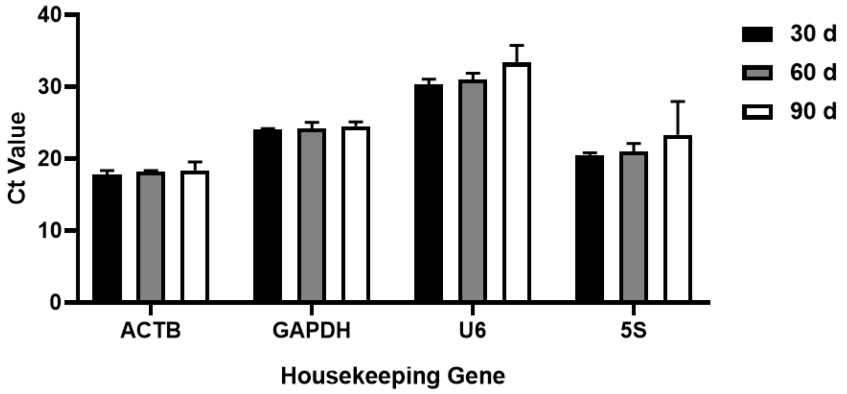

Figure S2. Ct values of four candidate housekeeping genes across testis sample at 30-90 d.

Table S2. The Ct values of four housekeeping genes during developmental stages of porcine testis.

Age	ACTB	GAPDH	U6	5S
30 d-1	18.66	23.73	30.91	20.02
	18.54	23.73	30.36	20.26
	18.17	24.90	31.26	19.82
30 d-2	17.26	23.55	N/A	21.08
	17.70	24.85	N/A	20.72
	17.31	23.31	N/A	20.59

	17.14	25.04	29.90	20.55
30 d-3	17.66	24.14	29.88	20.24
	17.31	23.27	29.87	20.51
	18.62	24.88	30.87	21.39
60 d-1	18.26	25.33	N/A	21.50
	18.18	25.12	30.28	21.14
	17.82	24.12	30.51	19.39
60 d-2	18.46	24.18	30.46	20.20
	18.19	23.87	30.49	19.84
	18.13	23.77	32.01	21.39
60 d-3	18.12	23.57	N/A	22.67
	18.27	22.97	N/A	21.76
	16.85	24.72	34.67	18.95
90 d-1	17.24	25.06	36.02	19.43
	17.27	24.64	35.16	18.89
	18.42	25.28	30.35	27.07
90 d-2	18.69	25.07	30.41	28.57
	18.55	25.30	31.27	29.26
	19.48	24.59	33.82	22.50
90 d-3	19.19	24.14	34.35	22.64
	19.70	23.70	34.10	22.41

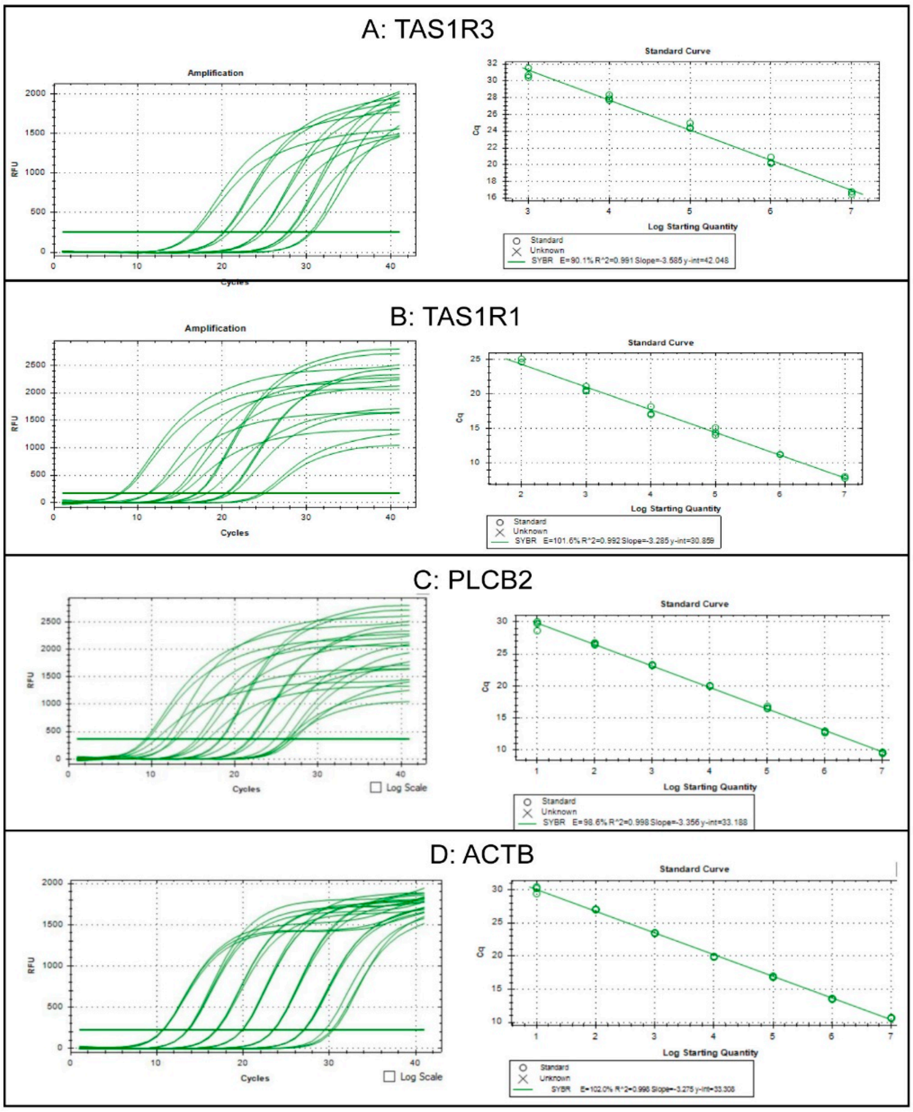

Figure S3. The amplification and standard curve of TAS1R3, TAS1R1, PLCB2 and ACTB in Q-PCR experiment. A, *TAS1R3* primer; B, *TAS1R1* primer; C, *PLCB2* primer; D, *ATCB* primer.

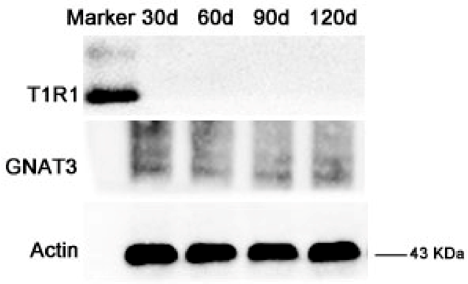

Figure S4. Expression of T1R1 and GNAT3 in testes of Congjiang Xiang pig at 30 d, 60 d, 90 d and 120 d of age. No specific band of T1R1 and GNAT3 proteins was detected in porcine testes by Western bloting.

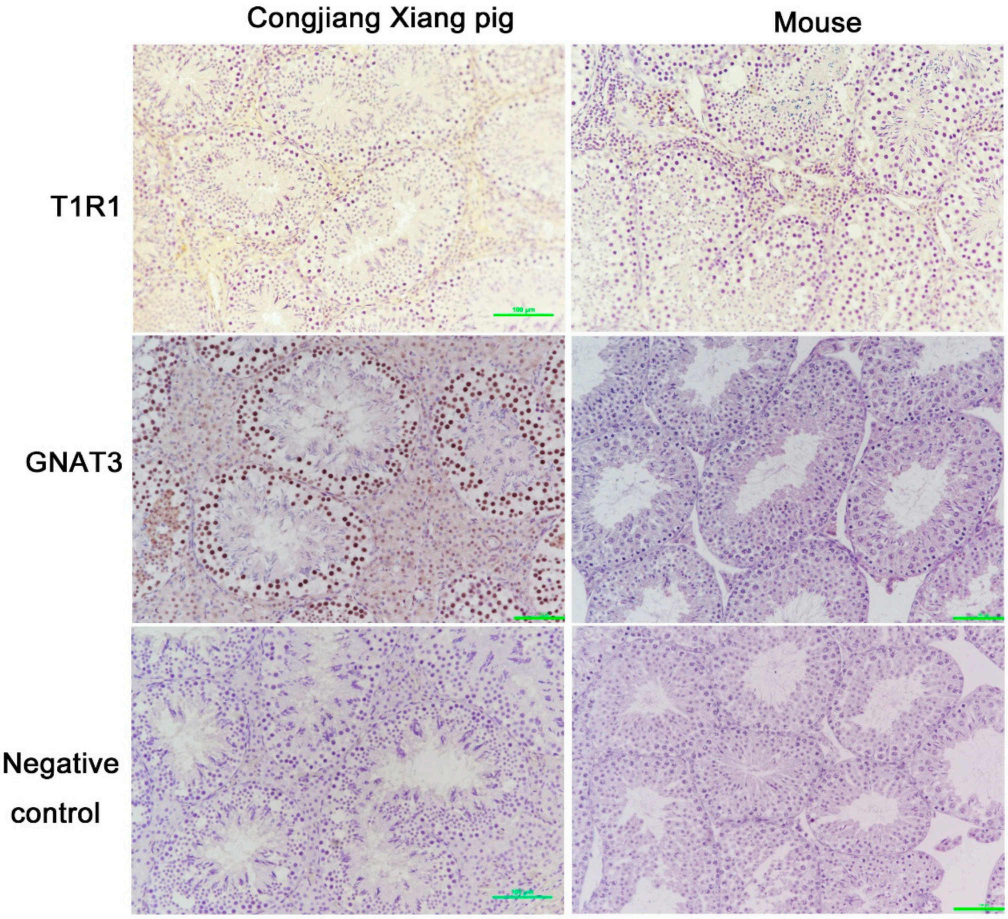

Figure S5. Immunolocalization of T1R1 and GANT3 proteins in testes of Congjiang Xiang pigs and mice. Sections were hardly immunoreacted with primary antibodies to T1R1 and GNAT3 both in Congjiang Xiang pig's tests (180 d, n=3) and mouse testes (60 d, n=3).

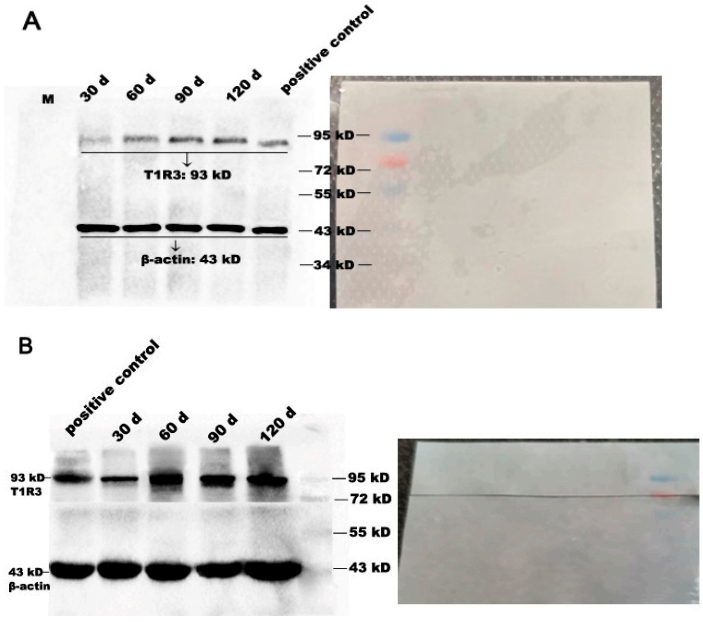

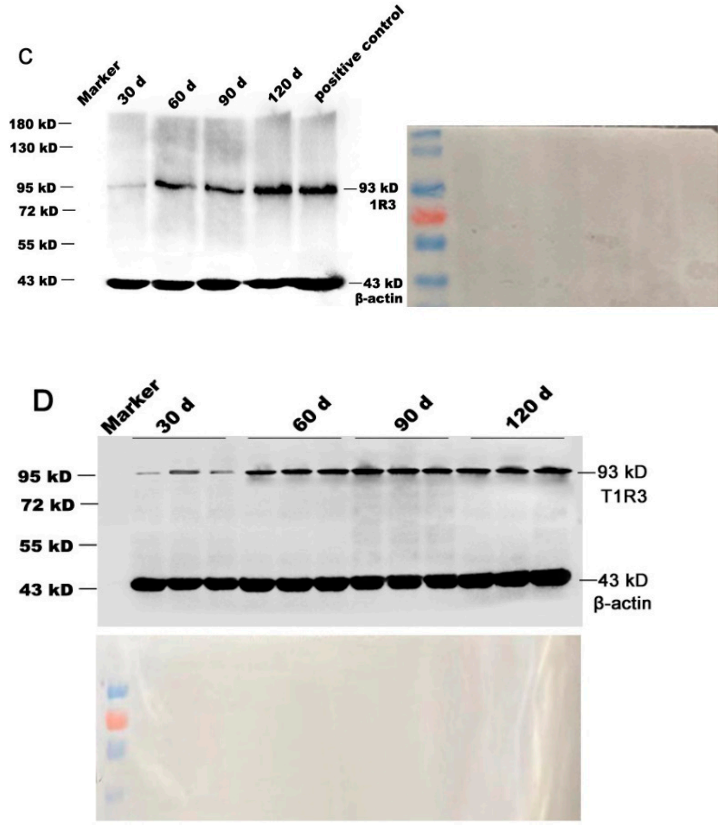

Figure S**6.** WB experiment of T1R3 antibody in the Congjiang Xiang pig testes at 30-120 d. The expression pattern of T1R3 was measured by WB, β-Actin was used as an internal control, the mouse testis (at 60 d) was severed as a positive control. A-C, three samples collected from three individual animals in each age (30 d, 60 d, 90 d and 120 d) were performed WB analysis in three blots, respectively. D, the samples at four age periods (n=3) were analyzed in one blot. Prestained color protein marker (P0068; Beyotime, Shanghai, China) was used to indicate the size of target proteins.

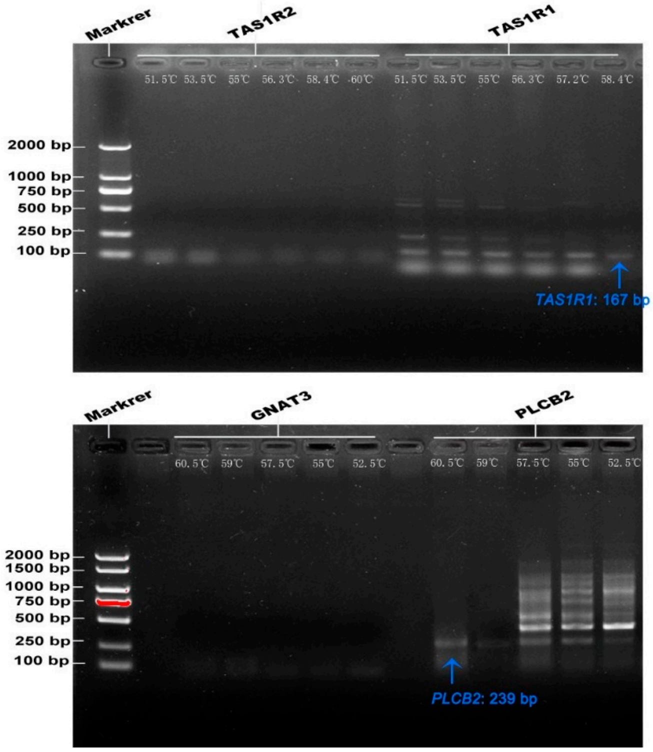

Figure S**7.** Transcription of *TAS1R1*, *TAS1R2*, *GNAT3* and *PLCB2* in the adult Congjiang Xiang pigs.Gel electrophoresis (1% Agarose, 100 V, 30 min) RT-PCR results showed *TAS1R1* and *PLCB2* expressed in testes of Congjiang Xiang pigs without *TAS1R2* and *GNAT3* expression (180 d, n=3). Marker: DL 2000 DNA marker.

Reference

1. Maseka, T.; Vopalenskya, V.; Suchomelovab, P.; Pospiseka, M. Denaturing RNA electrophoresis in TAE agarose gels. *Anal. Biochem.* 2005, 336, 46-50.
2. Fleige, S.; Pfaffl, M.W. RNA integrity and the effect on the real-time qRT-PCR performance. *Mol. Aspects Med.* 2006, 27, 126-139.
